# Identification and Comparison of Bioactive Components of Two *Dryopteris* sp. Extract Using LC-QTOF-MS

**DOI:** 10.3390/plants11233233

**Published:** 2022-11-25

**Authors:** Yangseon Kim, Da Jung Lim, Jeong-Sup Song, Jung-Ae Kim, Byoung-Hee Lee, Youn Kyoung Son

**Affiliations:** 1 Department of Research and Development, Center for Industrialization of Agricultural and Livestock Microorganisms, Jeongeup-si 56212, Republic of Korea; 2 Biological and Genetic Resources Assessment Division, National Institute of Biological Resources, Incheon 22689, Republic of Korea

**Keywords:** *Dryopteris* species, flavonoids, terpenoids, antibacterial activity, Gram-positive bacteria

## Abstract

*Dryopteris* sp. is known for its various pharmacological effects and is used as a traditional medicine in Asia. The present study investigated the chemical composition and antimicrobial activity of *Dryopteris* sp. distributed in Korea. The chemical compounds in the ethanolic extracts of *Dryopteris lacera* and *Dryopteris bissetiana* were investigated by ultra-high performance liquid chromatography–quadrupole time-of-flight–mass spectrometry analysis and identified by exploring the UNIFI traditional medicine library. Flavonoids such as juglanin, 6-hydroxyluteolin 7-O-laminaribioside, peltatoside, kaempferitrin, hyperoside, and astragalin were identified in both *D. lacera* and *D. bissetiana*. Neochlorogenic acid was identified as a caffeoylquinic acid in *D. bissetiana*. Both extracts of *D. lacera* and *D. bissetiana* exhibited antibacterial activity against Gram-positive pathogens, *Staphylococcus aureus* and *Streptococcus mutans*. The minimum inhibitory concentration of *D. bissetiana* against *S. aureus* was less than 625 ppm. The antibacterial activity was attributed to the identified phenolic compounds, juglanin, 6-hydroxyluteolin 7-O-laminaribioside, kaempferitrin, astragalin, and neochlorogenic acid. Therefore, *D. lacera* and *D. bissetiana* can be used as Gram-positive selective antibiotics for further investigation.

## 1. Introduction

Flavonoids are an important group of natural products with polyphenolic structures. They belong to a class of plant secondary metabolites widely found in fruits, vegetables, grains, flowers, tea, and wine. Flavonoids demonstrate various biological activities in plants, animals, and bacteria. These natural compounds protect plants from different biotic and abiotic stresses and play functional roles as signal molecules, antimicrobial defensive compounds, and resistance to environmental conditions that are harmful to growth [1]. In particular, flavonoids have beneficial effects on human and animal health. They are considered essential components in several nutraceutical, pharmaceutical, medicinal, and cosmetic applications due to their anti-oxidative, anti-inflammatory, anti-mutagenic, and anti-cancer properties, combined with their ability to modulate critical cellular enzyme functions [2]. Recently, antibiotic resistance has emerged as a major global problem, and new therapeutic agents are urgently required. Among many studies, flavonoids in the field of anti-infective compounds have been reported to exhibit antibacterial, antiviral, and antifungal properties [3,4]. Furthermore, flavonoids are natural compounds extracted from plants and have potentially available activities such as direct antibacterial activity, synergistic effects with antibiotics, and inhibition of bacterial toxicity. Therefore, flavonoids can be effective in the treatment and prevention of various infectious and toxic-mediated diseases.

Terpenoids, also known as isoprenoids or terpenes, are generally considered plant or fungal metabolites and are a large class of natural products ubiquitous in nature. Terpenoids have ring structures and exhibit a wide range of biological activities. The biological activity of certain terpenoids is often assumed to interact with their membrane-binding proteins or to be lipophilic. Aromatic plants and wood resins, such as turpentine, produce terpenoids that are the main ingredient of essential oils. Moreover, many plants containing terpenoids are used in traditional medicine because of their anti-inflammatory and pain-relieving properties [5]. Several species belonging to the Asteraceae family have been traditionally used to treat inflammatory conditions. These plants produce sesquiterpene lactones that contribute to their therapeutic activities [6]. Many essential oils have been tested for their anti-inflammatory and analgesic activities in various cellular and in vivo animal models. A recent study has also reported the anti-diabetic properties of terpenoids because of their ability to lower blood glucose levels by regulating glucose metabolism in humans and animals. In addition, they have a range of biological activities, including antimalarial and antimicrobial activities [7,8,9]. There are approximately 200 types of triterpenoids with different structural characteristics, a majority of which have antiviral, antitumor, antioxidant, and anti-inflammatory activities [10,11].

*Dryopteris* is a perennial herb distributed worldwide, which is estimated to contain 300 species with the highest diversity in eastern Asia [12,13,14]. *Dryopteris lacera* (Thunb.) Kuntze and *Dryopteris bissetiana* (Baker) C. Chr. are among the plants that belong to the *Dryopteris* genus of the Dryopteridaceae family. The extract of *Dryopteris* sp. has been reported to have antimicrobial [15], cytoprotective [16], antioxidant [17], and anticancer effects [18], and has been used as a traditional medicine in Asia [19]. Moreover, the rhizome of *Dryopteris* sp. has been reported to contain various chemical compounds such as phloroglucinols, flavonoids, and triterpenes [20].

In this study, we investigated the chemical compounds, flavonoids and terpenoids, of *D. lacera* and *D. bissetiana* through ultra-high-performance liquid chromatography– quadrupole time-of-flight–mass spectrometry (UPLC–QTOF–MS) analysis. The components were identified the UNIFI traditional medicine library. Additionally, the antimicrobial activity of the ethanolic extracts of *D. lacera* and *D. bissetiana* against Gram-positive and Gram-negative bacteria and fungal pathogens was performed.

## 2. Results and Discussion

**Figure 1:**
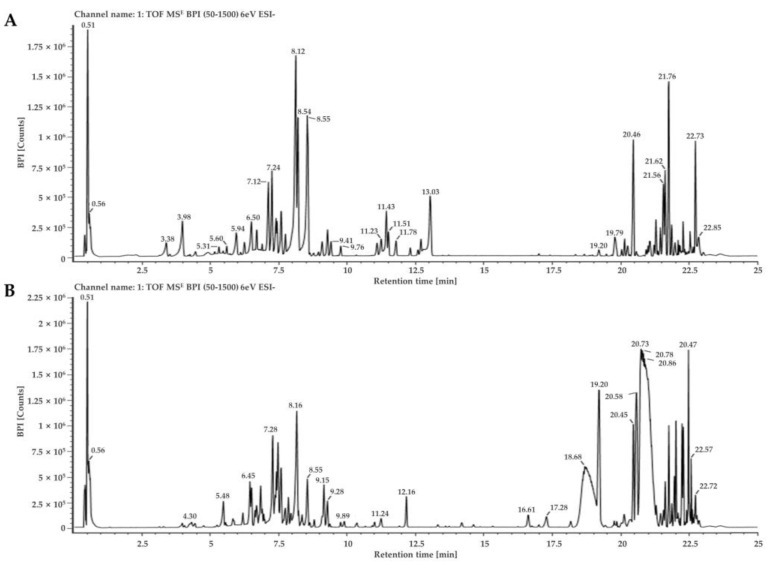
Base peak ion(BPI) chromatogram of *D. lacera* (**A**), and *D. bissetiana* (**B**) extracts by UPLC-QTOF-MS analysis.

**Figure 2:**
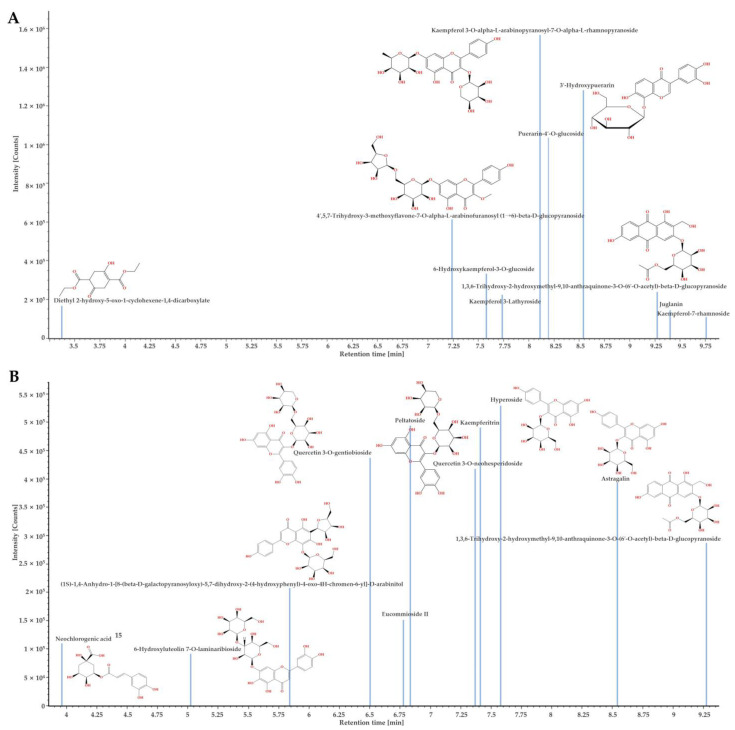
The confirmed component plot of UPLC-QTOF-MS analysis of *D. lacera* (**A**), and *D. bissetiana* (**B**) extracts.

The bioactive components of *D. lacera* and *D. bissetiana* were identified by UPLC–QTOF–MS analysis. The natural product analysis was performed using the LC–QTOF MS^E^ mode. In this mode, the main precursor ion was detected using a low collision energy value, which was then exposed to stronger collision energy to analyze the pattern of the product ions. Figure 1 and Figure 2 shows the UPLC-QTOF-MS base peak ion (BPI) chromatogram and component confirmed plot chromatogram. The relevant data, including retention time (min), experimental *m/z* [M−H]^−^ or [M+HCOO]^−^, theoretical mass, predicted chemical formula, and tentatively identified compound are listed in Table 1 and Table 2. The retention times, mass spectra, and fragment information were compared, and compounds were identified by exploring the UNIFI traditional medicine library containing over 600 herbs and over 6000 compounds. Therefore, it was possible to identify flavonoids and terpenoids compounds containing glucose.

**Figure 3:**
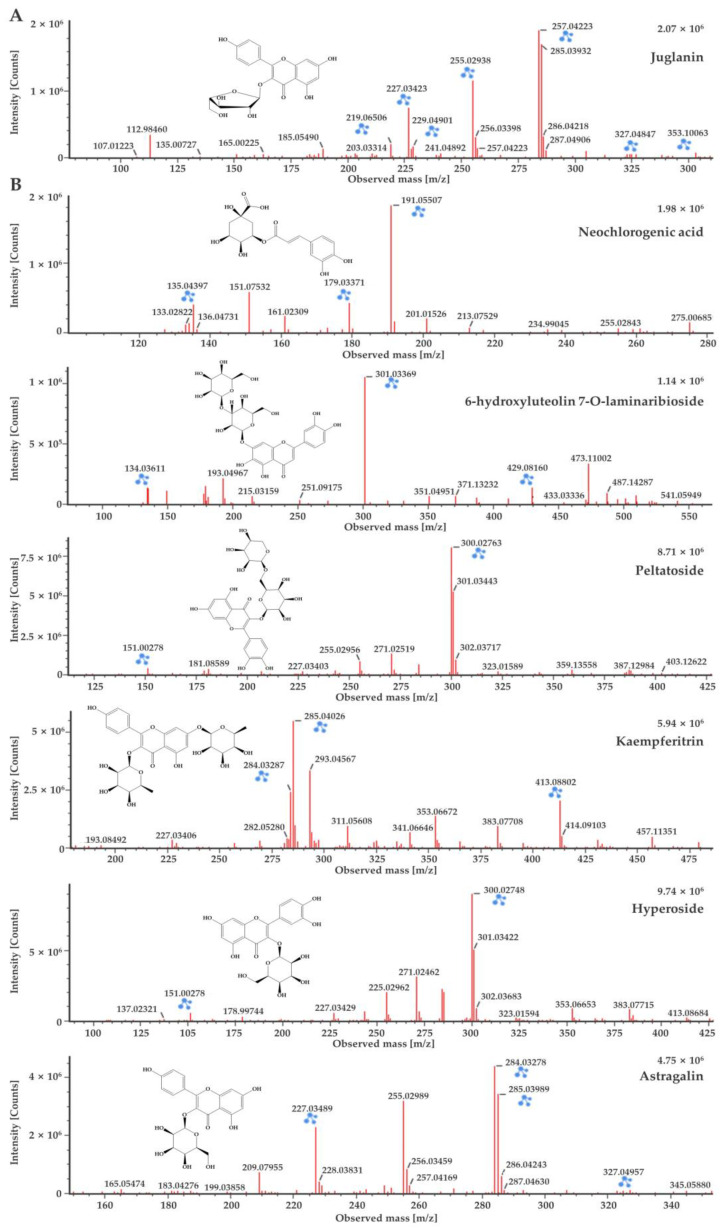
UPLC-QTOF-MS analysis of the major components in terms of matching scores of *D. lacera* (**A**), and *D. bissetiana* (**B**) extracts.

The chemical compound determined from the *D. lacera* UPLC–QTOF–MS analysis at a retention time of 9.4 min was identified as juglanin, which presented [M−H]^−^ ion at *m/z* 417. The product ions were formed at *m*/*z* 284 and *m*/*z* 255, with the main peak at *m*/*z* 284 attributed to the loss of glucose moiety (Figure 3A). Juglanin is a natural product found in many plants. It has various biological activities, including antimicrobial, anticancer, antioxidant, and anti-inflammatory effects, as well as protecting skin from ultraviolet B exposure [21,22,23,24,25]. 

Flavonoids, 6-hydroxyluteolin 7-O-laminaribioside, peltatoside, kaempferitrin, hyperoside, and astragalin from *D. bissetianai* were detected by UPLC–QTOF–MS analysis (Table 2). The [M−H]^−^ ion at *m*/*z* 625 with two glucose moieties attached was identified as 6-hydroxyluteolin 7-O-laminaribioside. The fragment ions were formed at *m*/*z* 429 without glucose moieties and at *m*/*z* 301 with only one glucose moiety. The main peak at *m*/*z* 301 indicated the loss of two glucose moieties. Peltatoside was identified at *m*/*z* 595 [M−H]^−^, with its fragment ions at *m*/*z* 300 and *m*/*z* 151. The main peak at *m*/*z* 300 was suggested the structure without the glucose moiety. Kaempferitrin confirmed at *m*/*z* 577 [M−H]^−^, with its fragment ions at *m*/*z* 413 and *m*/*z* 285. The main peak at *m*/*z* 285 was formed due to the loss of the glucose moiety. Hyperoside was detected at *m*/*z* 463 [M−H]^−^, with fragment ions at *m*/*z* 300 and *m*/*z* 151. The main peak at *m*/*z* 300 indicated the loss of glucose moiety. Astragalin was determined at *m*/*z* 447 [M−H]^−^, and the fragment ions were confirmed at *m*/*z* 284 and *m*/*z* 227. The main peak at *m*/*z* 284 suggested the loss of glucose moiety (Figure 3B). Phenolic compounds, including 6-hydroxyluteolin 7-O-laminaribioside of the medicinal plant *Globularia alypum*, exhibits antioxidant and antimicrobial activities [26]. Peltatoside isolated from *Annona crassilora*, used in folk medicine, is known to inhibit acute local inflammation and demonstrate analgesic activity [27,28]. Kaempferitrin from many plants shows antimicrobial, antitumor, and antioxidant activities [29,30,31]. It has also been suggested as a putative therapeutic agent for diabetic nephropathy by suppressing the mitochondrial/cytochrome c-mediated apoptosis pathway [32] and rheumatoid arthritis (RA) by ameliorating inflammation in RA fibroblast-like synoviocytes [33]. Hyperoside is a quercetin 3-d-galactoside derived from plants that is known to have anti-cancer effects. It inhibits the cervical cancer HeLa and C-33A cell proliferation [34], A549 and H1975 lung cancer cell proliferation [35], and induces apoptosis in HT-29 colon [36], SKOV3 and HO-8910 ovarian [37], and MCF-7 and 4T1 breast cancer cells [38]. Astragalin is kaempferol-3-O-ß-D-glucoside, a bioactive natural flavonoid that is known for its medicinal importance such as antimicrobial, antioxidant, anti-inflammatory, anti-cancer, and neuroprotective activities [39,40,41,42,43,44]. It is also applied in cosmetics to inhibit melanin secretion and protect against UV damage [45,46]. Neochlorogenic acid was identified as a caffeoylquinic acid in *D. bissetiana* (Table 2) at *m*/*z* 353 [M−H]^−^. The fragments ions were confirmed at *m*/*z* 191 and *m*/*z* 179. The main peak at *m*/*z* 191 suggested the loss of the caffeic acid group, while the peak at *m*/*z* 179 indicated the loss of the quinic acid group (Figure 3B). Neochlorogenic acid is a natural polyphenolic compound found in plants, known for its antioxidant, antibacterial, antitumor, and anti-inflammatory properties [47,48]. Furthermore, neochlorogenic acid shows anti-photoaging effects and is an effective agent for skin wrinkle formation and dehydration [49]. 

The antibacterial assay revealed that *D. lacera* and *D. bissetiana* exhibits antibacterial activity against the selected indicator pathogenic strains, as summarized in Table 3. A clear zone of inhibition of *S. aureus* NCCP14560 and *S. mutans* KCTC3065 was observed using *D. lacera* and *D. bissetiana* extracts. The MIC of *D. lacera* extract was 5 mg/mL against *S. aureus* and *S. mutans*, and the MIC of *D. bissetiana* extract was 0.625 mg/mL and 5 mg/mL against each pathogen, respectively (Table 3). Conversely, *D. lacera* and *D. bissetiana* extracts were inactive against *E. coli* KCTC2617, *S.* Enteritidis NCCP14546, and *C. albicans* NCCP31077. Phenolic compounds identified in *D. lacera* and *D. bissetiana* are known for their antimicrobial activity. It has been reported that ethanol extracts of plants contain various chemical compounds that affect multiple sites of bacterial cell walls [50]. However, the ethanolic extracts of *D. lacera* and *D. bissetiana* only had antimicrobial effects on *S. aureus* and *S. mutans*, which are Gram-positive bacterial pathogens among the tested microorganisms. Moreover, the *D. bissetiana* extract showed antibacterial activity against *S. aureus* at very low concentrations of less than 0.625 mg/mL suggesting further study of its clinical use. In particular, the emergence of antibiotic-resistant strains of *S. aureus*, such as methicillin-resistant *S. aureus* and vancomycin-resistant *S. aureus*, is a global risk in clinical medicine.

## 3. Materials and Methods

### 3.1. Plant Materials and Preparation of Plant Extract

The leaves of *D. lacera* (Thunb.) Kuntze and *D. bissetiana* (Baker) C. Chr were collected from Mt. Jiri of Gyeongsang-do, South Korea, and identified by an experienced taxonomist. The collected specimens of *D. lacera* Kuntze and *D. bissetiana* C. Chr were deposited in the Natural Products Bank, Wildlife Genetic Resources Center at the National Institute of Biological Resources. The collected leaves were dried in a drying oven at 40 °C for 2 d for preparing the crude extract. Approximately 500 g of dried leaves were extracted from 70% ethanol (analytical grade, Sigma-Aldrich, St. Louis, MI, USA), four times the volume of the leaves, for 48 h. The ethanol crude extracts from the leaves were centrifuged at 4000× *g* for 5 min, filtered using a Bückner funnel, and all ethanol was removed using a rotary evaporator. The extract was stored at −80 °C in a freezer until further use.

### 3.2. UPLC-QTOF-MS Analysis of Dryopteris Plants

The chemical profile of the 70 % EtOH extract of plants was analyzed using a Waters ACQUITY UPLC I-Class PLUS equipped with a Waters ACQUITY UPCL HSS T3 column (100 mm × 2.1 mm, 1.8 µM), maintained at an isothermal temperature of 40 °C. A binary pump delivered the mobile phase at flow rate of 0.2 mL/min under a gradient elution using two mobile phases, water containing 0.1 % (*v*/*v*) formic acid (solvent A) and acetonitrile containing 0.1 % (*v*/*v*) formic acid (solvent B). The following were the gradient conditions: 0–1 min, 95 % solvent A; 1–17 min, 60 % solvent A; 17–21 min, 40 % solvent A; 11–22 min, 0 % solvent A; 22.9–25 min, 95 % solvent A. The auto-sampler was set to an injection volume of 5 µL. Mass spectrometric analysis was performed using a Waters Xevo-G2-XS QTOF LCMS equipped with an electrospray ionization source. The analysis was conducted in negative ion mode, set for detecting mass-to-charge ratio (*m*/*z*) in the range of 50–1500. The source temperature was set at 120 °C with a capillary voltage 2.5 kV. Ar (g) was used as the collision gas. Data acquisition and analysis were controlled using the traditional medicine library of Waters’ UNIFI software 1.9 version. In addition, the identification of the components was performed by checking the quality allowable error range ± 5 ppm fragmentation pattern. If the fragment ion pattern did not match, it was finally identified using a Chemspider (http://www.chemspider.com/, accessed on 5 November 2021) online database. 

### 3.3. Antibacterial Analysis

The antibacterial activities of plant extracts against well-known pathogenic microorganisms were evaluated using a previously described disk diffusion method [51], with slight modifications. The following five microbial pathogens were used as indicators of antibacterial activities: *E. coli* KCTC2617, *Salmonella enterica* serovar Enteritidis NCCP14546, *Staphylococcus aureus* NCCP14560, *Streptococcus mutans* KCTC3065, and *Candida albicans* NCCP31077. Initially, these pathogenic strains were grown on suitable media at 30–37 °C for 20 h. *E. coli* and *S. aureus* were grown on nutrient agar, *S. Enteritidis* on tryptic soy agar, *S. mutans* on brain heart infusion agar, and *C. albicans* on Sabouraud dextrose agar. Diffusion disks with a diameter of 8 mm were placed on the agar, and the plant extract with a concentration of 20 mg/mL using a diluent solvent (DMSO:EtOH = 1:1, *v*/*v*) was dispensed onto the disks. The plates were incubated at 30–37 °C for 24 h, and the diameters of the inhibition zones around each disk were measured for positive and negative controls, gentamycin (2.5 mg/mL), hygromycin (10 mg/mL), and a diluent solvent were used as, respectively. Further, different concentrations of plant extract (20, 5, 2.5, 1.25, and 0.625 mg/mL were prepared by five-fold serial dilution to determine the minimum inhibitory concentrations (MIC). MIC is regarded as the lowest concentration that inhibits the microbial pathogen growth. Each prepared extract was applied to pathogen-inoculated agar media and then incubated under the required growth conditions [52]. All antimicrobial assays were conducted in triplicates.

### 3.4. Statistical Analysis

Data were analyzed using One-way ANOVA Tukey post hoc test for multiple comparisons by IBS SPSS Statistics 20 software. All experiments were conducted in three replicates per treatment.

## 4. Conclusions

In conclusion, we identified the chemical compounds of *D. lacera* and *D. bissetiana* by UPLC–QTOF–MS and presented various biological phenolic compounds, such as juglanin, 6-hydroxyluteolin 7-O-laminaribioside, peltatoside, kaempferitrin, hyperoside, astragalin, and neochlorogenic acid. Both *Dryopteris* plants in this study showed antibacterial activity against the Gram-positive pathogens. However, the extracts of *D. lacera* and *D. bissetiana* did not exhibit antimicrobial activity against the Gram-negative. Therefore, *D. lacera* and *D. bissetiana* can be used as Gram-positive selective antibiotics for further investigation.

## Figures and Tables

**Table 1:** The bioactive components of *D. lacera* based on the UPLC-QTOF-MS analysis.

RT (min)	Experimental *m*/*z* [M−H]^−^ or [M+HCOO]^−^	Theoretical Mass	Mass Error (ppm)	Molecular Formula	Tentatively Identified Compound	Metabolite Class
3.37	255.0876	210.08921	0.7	C11H14O4	Diethyl 2-hydroxy-5-oxo-1-cyclohexene-1,4-dicarboxylate	Terpenoid
7.24	593.152	594.15847	1.4	C27H30O15	4′,5,7-Trihydroxy-3-methoxyflavone-7-O-alpha-L-arabinofuranosyl (1→6)-beta-D-glucopyranoside	Flavonoid
7.58	463.0883	464.09548	0.2	C21H20O12	6-Hydroxykaempferol-3-O-glucoside	Flavonoid
7.74	579.1364	580.14282	1.5	C26H28O15	Kaempferol 3-Lathyroside	Flavonoid
8.11	563.141	564.14791	0.7	C26H28O14	Kaempferol 3-O-alpha-L-arabinopyranosyl-7-O-alpha-L-rhamnopyranoside	Flavonoid
8.19	577.1566	578.16356	0.5	C27H30O14	Puerarin-4′-O-glucoside	Flavonoid
8.54	447.093	448.10056	−0.5	C21H20O11	3′-Hydroxypuerarin	Flavonoid
9.27	489.1036	490.11113	−0.4	C23H22O12	1,3,6-Trihydroxy-2-hydroxymethyl-9,10-anthraquinone-3-O-(6′-O-acetyl)-beta-D-glucopyranoside	Quinone
9.4	417.0826	418.09000	−0.3	C20H18O10	Juglanin	Flavonoid
9.76	431.0983	432.10565	−0.1	C21H20O10	Kaempferol-7-rhamnoside	Flavonoid

**Table 2:** The bioactive components of *D. bissetiane* based on the UPLC-QTOF-MS analysis.

RT (min)	Experimental *m*/*z* [M−H]^−^ or [M+HCOO]^−^	Theoretical Mass	Mass Error (ppm)	Molecular Formula	Tentatively Identified Compound	Metabolite Class
3.96	353.0873	354.09508	−1.5	C16H18O9	Neochlorogenic acid	Caffeoylquinic acid
5.02	625.1402	626.1483	−1.3	C27H30O17	6-Hydroxyluteolin 7-O-laminaribioside	Flavonoid
5.84	579.1371	580.14282	2.7	C26H28O15	(1S)-1,4-Anhydro-1-[8-(beta-D-galactopyranosyloxy)-5,7-dihydroxy-2-(4-hydroxyphenyl)-4-oxo-4H-chromen-6-yl]-D-arabinitol	Flavonoid
6.50	625.1413	626.1483	0.5	C27H30O17	Quercetin 3-O-gentiobioside	Flavonoid
6.78	349.1505	350.15768	0.3	C15H26O9	Eucommioside II	Terpenoid
6.84	595.1307	596.13773	0.4	C26H28O16	Peltatoside	Flavonoid
7.37	609.1462	610.15338	0.2	C27H30O16	Quercetin 3-O-neohesperidoside	Flavonoid
7.41	577.1565	578.16356	0.4	C27H30O14	Kaempferitrin	Flavonoid
7.58	463.0881	464.09548	−0.2	C21H20O12	Hyperoside	Flavonoid
8.54	447.0936	448.10056	0.8	C21H20O11	Astragalin	Flavonoid
9.27	489.1044	490.11113	1.2	C23H22O12	1,3,6-Trihydroxy-2-hydroxymethyl-9,10-anthraquinone-3-O-(6′-O-acetyl)-beta-D-glucopyranoside	Quinone

**Table 3:** Antibacterial activity of the plant extract against the indicator strains.

Test Pathogenic Strain	Antibacterial Activity					
	*D. lacera*		*D. bissetiana*		Positive Control	
	Inhibition ^a^	MIC (mg/mL)	Inhibition	MIC (mg/mL)	Antibiotics	Inhibition
*Escherichia coli* KCTC2617	-		-		Gentamycin	++
*Salmonella enterica* serovar Enteritidis NCCP14546	-		-		Gentamycin	++
*Staphylococcus aureus* NCCP14560	+++	5	+++	0.625	Gentamycin	++
*Streptococcus mutans* KCTC 3065	+++	5	+++	5	Gentamycin	++
*Candida albicans* NCCP31077	-		-		Hygomycin	++

^a^ The inhibition zone (mm) around the paper disc containing the microbial cell-free supernatant was classified as +++, >13 mm; ++, 10–12 mm; +, less than 9 mm; -, no inhibition zone. All microbial pathogens showed no inhibition against the negative control (DMSO:EtOH = 1:1, *v*/*v*).

## Data Availability

Not applicable.
